# Vehicle Speed and Length Estimation Using Data from Two Anisotropic Magneto-Resistive (AMR) Sensors

**DOI:** 10.3390/s17081778

**Published:** 2017-08-03

**Authors:** Vytautas Markevicius, Dangirutis Navikas, Adam Idzkowski, Algimantas Valinevicius, Mindaugas Zilys, Darius Andriukaitis

**Affiliations:** 1 Department of Electronics Engineering, Kaunas University of Technology, Studentu St. 50–418, LT-51368 Kaunas, Lithuania; dangirutis.navikas@ktu.lt (D.N.); a.idzkowski@pb.edu.pl (A.I.);algimantas.valinevicius@ktu.lt (A.V.); mindaugas.zilys@ktu.lt (M.Z.); darius.andriukaitis@ktu.lt (D.A.); 2 Faculty of Electrical Engineering, Bialystok University of Technology; Wiejska St. 45D, Bialystok PL-15351, Poland

**Keywords:** magnetic field, AMR sensors, vehicle speed detection, car length estimation

## Abstract

Methods for estimating a car’s length are presented in this paper, as well as the results achieved by using a self-designed system equipped with two anisotropic magneto-resistive (AMR) sensors, which were placed on a road lane. The purpose of the research was to compare the lengths of mid-size cars, i.e., family cars (hatchbacks), saloons (sedans), station wagons and SUVs. Four methods were used in the research: a simple threshold based method, a threshold method based on moving average and standard deviation, a two-extreme-peak detection method and a method based on the amplitude and time normalization using linear extrapolation (or interpolation). The results were achieved by analyzing changes in the magnitude and in the absolute z-component of the magnetic field as well. The tests, which were performed in four different Earth directions, show differences in the values of estimated lengths. The magnitude-based results in the case when cars drove from the South to the North direction were even up to 1.2 m higher than the other results achieved using the threshold methods. Smaller differences in lengths were observed when the distances were measured between two extreme peaks in the car magnetic signatures. The results were summarized in tables and the errors of estimated lengths were presented. The maximal errors, related to real lengths, were up to 22%.

## 1. Introduction

Modern vehicle monitoring and traffic monitoring systems collect data concerning the flow of vehicles, speed and traffic conditions while processing this the data in real time. Methods and devices used to detect individual vehicles or entire car queues are the integral parts of such systems. A number of methods have appeared in the literature which are currently used to detect vehicles. However, a completely accurate vehicle-type-detection method has not been presented in the literature due to the variety in vehicle construction. The problem with a proper detection of a vehicle type is dependent on accurate speed measurements because speeds can be very different in traffic. Vehicle detection equipment uses technologies such as inductive loop detectors [1], video image processing systems [2], microwave radars [3], and active infrared detectors [4].

Recently, a lot of research has been carried out using anisotropic magneto-resistive (AMR) magnetic field sensors. The Earth’s magnetic field intensity is relatively weak, equaling 0.25–0.65 G, but a car that moves around the sensor area, causes measurable changes. The strength of the magnetic field, measured by a sensor, vary not only as a result of the car’s arrival and movement above sensors, but there can be some interferences due to any other massive metal objects in the area of the sensor such as an armature, road structure elements, etc. Detection efficiency is higher when the magnetic field is not disturbed by undesirable objects, which are not always possible to eliminate. Another flaw of AMR sensors is the strong parameter dependence on the ambient temperature. It is precisely for this reason that a vehicle detection with the use of AMR magnetic field sensors is more complicated compared to, e.g., inductive loops. However, it is possible to identify a type of vehicle [5].

Small and inexpensive 3-axis AMR magnetic field sensors are used in vehicle detection and classification systems. In contrast to Hall sensors, AMR sensors detect horizontal magnetic fields and have a wider detection area [6,7]. The use of two sensors allows one to measure speed, indicate the direction of motion and measure the length of a vehicle [8]. The purpose of this article is to present different methods for vehicle length estimation, and to compare the results achieved by means of these different methods.

## 2. Related Works

The first articles on this topic include methods for measuring the speed and length of vehicles by the use of dual-loop detectors and video analysis. Another way is the use of a single inductive loop detector that requires a speed measurement and then to calculate a product of that speed and time in which the sensor is active. Speed is determined by numerous trials of different vehicle lengths, statistical parameters (e.g., median) and frequency distribution histograms [1].

The wireless vehicle detection systems using AMR magnetic field sensors are presented in the following works: the work described in [5] concerns the use of two linear arrays with 16 sensors set at a distance of 3 m and a multi-scale wavelet discontinuity detection method for determining the start and the end of a magnetic vehicle signature. This method bases on the quadratic spline wavelet transform and low-pass and high-pass filtering. The magnitude of magnetic induction is calculated from three directional components *x*, *y*, *z*. The signature origin is defined as the most prominent (positive) point of the magnitude discontinuity. The end of the signature is the most prominent negative point of the magnitude discontinuity. For the tested algorithms, the efficiency of classifying vehicles for 5 categories has reached a level of 88%.

The length of a vehicle was also evaluated using a threshold method with *k*-times standard deviation of the noise floor as the threshold value [8]. An RMS error of 0.65 m was obtained, and in 95% of measurements the error was not greater than 1.25 m, which are quite high values. However, it should be noted that the detection zones of sensors are wide, i.e., the changes in magnetic fields are registered a bit earlier and a bit later while having a car drive over the sensors. Therefore, it seems that a threshold value, which is equal to two standard deviations, aiming to detect the vehicle’s front and back is too low. The vehicle classification is based on the Dynamic Time Warping method, which compares the time signal with the standard car signatures in a database.

In [9] the condition whether the magnitude of a magnetic field compared to the baseline of both sensors was greater than a threshold for a certain period of time was tested. It was considered as a vehicle arrival or as a noise therefore only the *z*-component of magnetic field induction was taken for analysis. In [9,10] the average of the energy and number of hill-pattern peaks were used to classify vehicles, the accuracy of the classification was from 68% to 95%, depending on the vehicle class. The best results were obtained for motorcycles. The number of peaks in signatures ranged from 1 to 6.

Typically, several (four or five) vehicle classes are considered. The main problem with the identification of vehicles by means of magnetic signatures is the ambiguity between classes, and especially within a single class. Sedans have different magnetic signature shapes. Therefore, it is very important to determine the length of a vehicle as well as a distance between peaks in a signature [11]. In addition, the position of the sensors relative to the Earth's magnetic pole affects the individual components of magnetic induction [12], which must be considered when comparing signals received from different places on the road.

In one of the previous papers [13], the authors of this article analyzed 12 methods of speed measurement, and two of them, namely z-component cross-correlation and *Kz* criterion cross-correlation, were considered to be the most accurate. The average relative error of speed determination did not exceed 1.5% for sensors located at a distance of 2 m. However, measurements were made in static (laboratory) conditions, which allowed the components of the measured magnetic field to be scanned in the direction of x-axis with resolution 1 cm at a distance of 1 m for both sensors. This resulted in obtaining accurate signatures and measured values.

The cross-correlation method is used in this research to determine a vehicle speed in the case when sensors are located at a distance of 1 m. The speed measurement is needed to determine a vehicle length and its signature. The analysis of the literature shows that the determined length value can depend on, e.g., the method of speed measurement, the method of setting a threshold in a measured signal, the driving precision along the axis of the road where the sensors are situated, the signal to noise ratio, as well as external factors, e.g., a change in ambient temperature. Therefore, the authors decided to use various methods in order to analyze and summarize the results.

## 3. Measuring System

**Figure 1:**
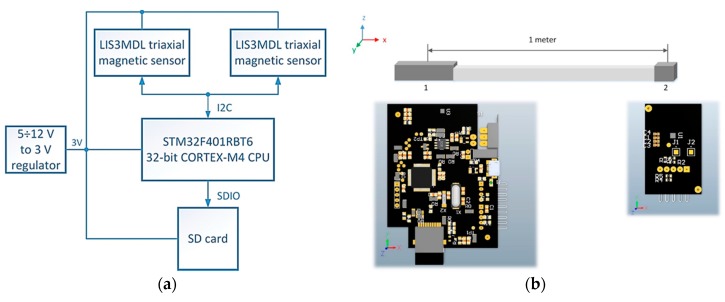
(**a**) The block scheme of the measuring system. (**b**) Developed PCBs for data acquisition, supplied from battery, connected by a wire and covered in plastic boxes.

The measuring system (Figure 1a) consists of two three-axis digital magnetic field sensors (LIS3MDL, STMicroelectronics Inc., Santa Clara, CA, USA), FSR = ±4 G, 6842 LSB/G. The 32-bit STM32F401RBT6 microcontroller (STMicroelectronics Inc., Santa Clara, CA, USA) is powered by a 3 V DC supply, with a sampling rate of 766 Hz. Communication between the microcontroller and the sensors is via I2C interface. RAW data from both sensors (representing the *x*, *y*, *z* components of the magnetic field induction) are recorded on the SD card.

The measuring system was constructed in the form of a flat rectangular shaped strip (Figure 1b). Inside the front end there is a PCB with a microcontroller, the first sensor and a SD card slot. The PCB with the second sensor is located inside the rear end of the strip. Both PCBs are connected by wires. This design ensures that the measuring system is adhered to the roadway.

## 4. Noise in Signals

**Figure 2:**
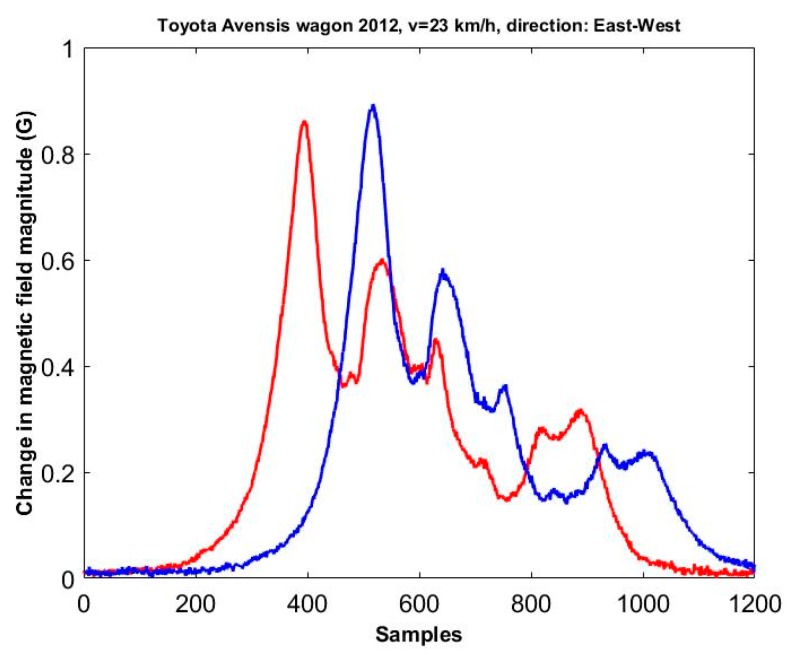
The changes in the magnetic field magnitude caused by Toyota Avensis that passed in the centre of a road line.

A signal represents a difference between the measured magnetic field and the magnetic field of an empty road lane. Exemplary time signals recorded by means of two sensors for a car (a 2012 Toyota Avensis wagon) traveling at 23 km/h are shown in Figure 2. These two signals are correlated and the correlation coefficient was 0.98. However, there are minor differences in the shape and value of peaks which occur in these signals. Many repeated observations indicate that the signals are never exactly the same.

**Figure 3:**
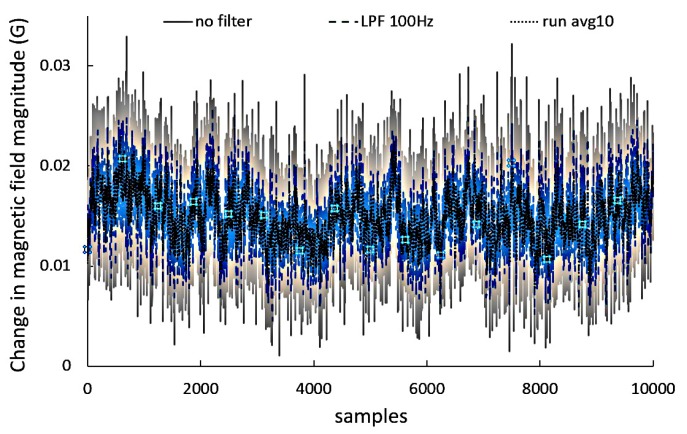
The change in the magnitude of the background magnetic field, noise and filtering effect.

Figure 3 shows a certain change in magnetic field background (mean is 15 mG) and a long-term noise. A threshold level in a recorded signal is required to get rid of the background noise and the interference from other objects. When the noise is too big, it is difficult to determine two points where the base line intersected with a signal line. More points on the base line can cause an incorrect measurement result. There are similarities during the detection of peaks in a signal.

A noised signal in comparison to a filtered one gives more peaks. Many vehicle detection algorithms include filtering steps, so several filtering methods were tested. The best effect was obtained for the running average.

Figure 3 shows a background signal without filtering (standard deviation SD = 4.4 mG), the same signal filtered with a two-pole low-pass filter with a 100 Hz cut-off frequency (SD = 3.2 mG) and the same signal filtered by running average 10 consecutive samples (SD = 2.5 mG). It is better to calculate the magnitude on the basis of *x*, *y*, *z* components of a magnetic field and then to filter this magnitude. As a result, we have less CPU arithmetic operations, the standard deviation is lower and the mean value of the signal is not changed.

## 5. Experiments

The system was used to measure speed and length of a running car at two different places in Lithuania (Kaunas and Kedainiai) and in four directions:

- from the North to the South (N-S) and from the South to the North (S-N)
- from the West to the East (W-E) and from the East to the West (E-W)

**Figure 4:**
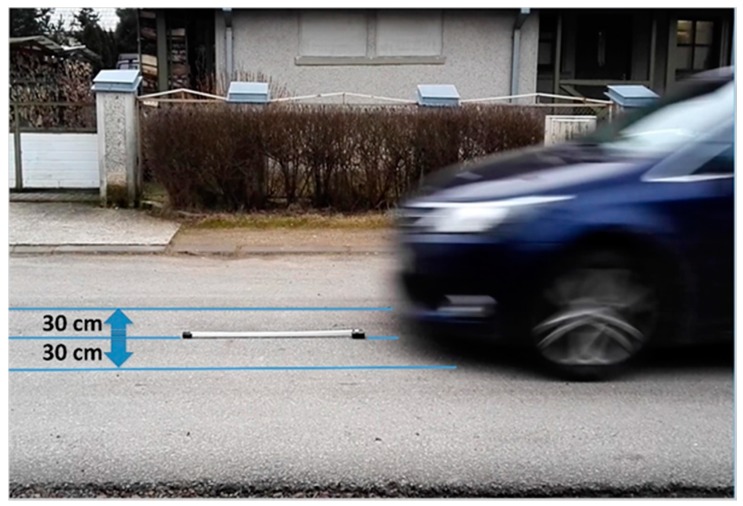
The measuring system placed on a road lane. A car is driven centrally (C) along a lane axis or non-centrally-with 30 cm shift to the right (R) or to the left (L).

The sensors were placed in the centre of a road lane (Figure 4). The passenger cars used (and their corresponding lengths) were: a 2012 Toyota Avensis wagon (4.765 m), 2008 Toyota Auris I hatchback (4.219 m), 2001Opel Vectra hatchback 2.2DTI (4.481 m), 2000 Volvo S60 sedan 2.4D (4.570 m), 2012 Skoda Superb II (4.838 m), 2009 Renault Coleos 2.0dCI (4.519 m) and a 2013 Lexus RX450 (4.770 m).

## 6. Speed and Length Measurement Evaluation on the Basis of the Magnitude Signal and Z-Component Signal of the Magnetic Field

On the basis of changes in the magnetic field strength, which is measured underneath a car, we can detect points when this car arrives at and departs from a detection area of two sensors. During this process the number of samples or the period of time are measured. However, this time depends on the amount of ferrous metal and the length of a car. Different cars drive with different speeds which are estimated as a result of the cross-correlation function of two signals. Algorithms described below and measurement methods were tested with the use of mid-size cars which weight 1173−2115 kg (unloaded).

### 6.1. Speed Measurement

The autocorrelation method was applied [13] and verified by a video camera (60 fps). Two sensors were placed at a distance of 1 meter. The short distance between the sensors solves the problem caused by doing any manoeuver, which may be detected, e.g., a lane change. Taking the video camera as a reference, the maximum error was 5% within the range 25−125 km/h.

The time delay in terms of samples is given by the formula:

(1) $$\Delta t=\Delta n\cdot t_{s}=\underset{n}{\arg\max}\;f\;[n]\cdot t_{s},$$

(2) $$f[n]=\sum_{m=0}^{N-1}B_{1}[m]\cdot B_{2}[m-n],\begin{array}{cc} & -(N-1)<n<N-1 \end{array},$$

where *B*_1_, *B*_2_ are the magnitudes of the magnetic field signal, N—number of samples, *n*—lag (a delay in samples), Δ*n*—sample delay, *t_s_*—sampling period. The highest peak of autocorrelation function *f* gives a lag in samples which is recalculated to time delay Δ*t*. If *B*_1_ and *B*_2_ are not the same length, the shorter vector is zero-padded to the length of the longer vector. The distance Δ*d* between sensors (1 m) and time delay Δ*t* equation is used to calculate speed *v*

(3) $$v=\frac{\Delta d}{\Delta t}.$$

### 6.2. The Simple Threshold-Based Detection Method (Method 1)

**Figure 5:**
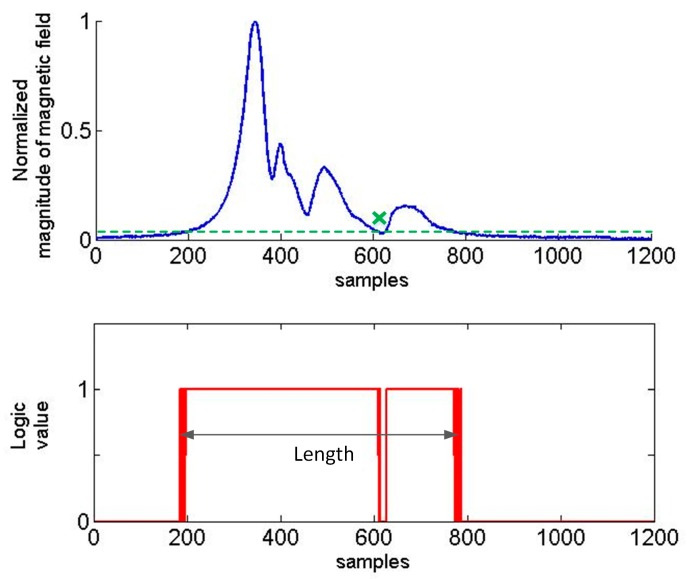
Toyota Auris—the magnitude signature length computation with a threshold level 0.035 G.

The simple threshold-based detection method to detect the vehicle presence is based on a user- defined threshold. If the magnitude (strength) of the measured signal is greater than a user-defined threshold, then a vehicle is determined as this one which passed. That is why the magnitude of a signal should be normalized (range from 0 to 1) and the threshold value as 0.05, 0.1 or any other value should be experimentally found for a selected car class. In the literature a 40 mG [14] or 60 mG [15] signal threshold was chosen to detect a car due to the interference induced by moving vehicles on the adjacent lanes. This threshold value cannot be too high because in the case of some cars (i.e., Toyota Auris) the normalized magnitude data points are near to 0–0.1 in the middle of time series (Figure 5). This led to the algorithm being more complicated.

### 6.3. Threshold Method Based on the Moving Average and kSD (Method 2)

**Figure 6:**
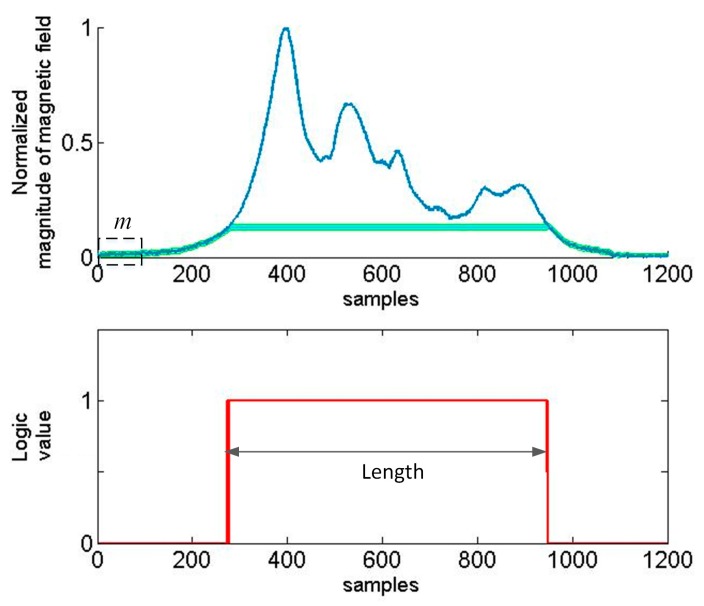
Toyota Avensis—the magnitude signature length computation with 3SD and running average of 100 samples.

The method is based on the moving average of a signal. The algorithm averages out the values of first *m* samples as the first data point, it adds another sample and takes another average. The standard deviation (SD) is calculated only one time (on the basis of first *m* = 10, 25 or 100 samples, number 10 gives a satisfactory effect, number 25 is optimal). Another important parameter is *n* which is also constant. It means if a data point is *k*-times standard deviation away from the moving average value then it is regarded as sudden peak in time series data. The length is defined as the number of samples when the curve in Figure 6 is over a threshold level.

### 6.4. Two-Extreme-Peak Detection (Method 3)

**Figure 7:**
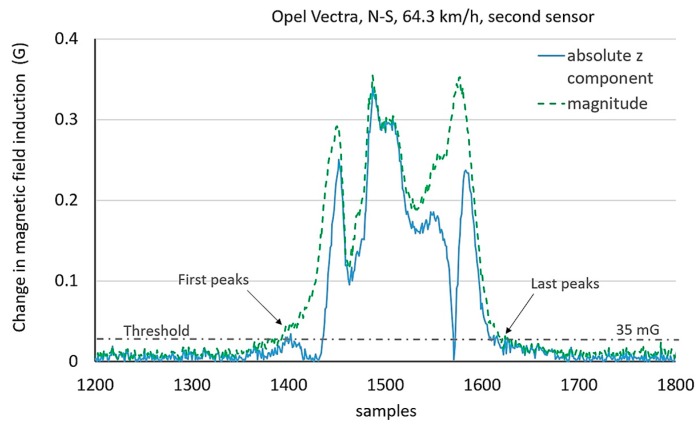
Problem of little peaks and too big threshold baseline considering absolute *z*-component and magnitude of magnetic field signal.

**Figure 8:**
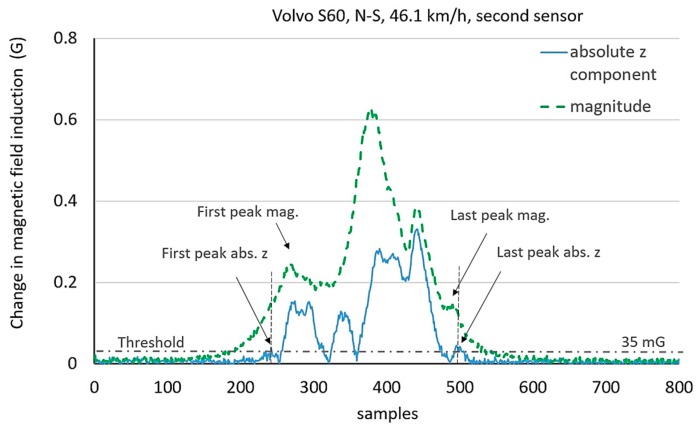
The problem of different numbers and positions of peaks considering the absolute z-component and the magnitude of magnetic field induction.

Threshold-based methods are used to determine the presence of a car on a roadway. It is impossible to precisely determine the length of a car by these methods. Therefore, the additional detection of some distinctive points in time series is needed, i.e., peaks. In the filtered signal there are usually several such peaks. Figure 5 shows four peaks, and Figure 6 shows six peaks in the signal of magnitude. The method described in this paper concerns the detection of two extreme peaks above a user defined value of threshold. The problem occurs if there are flat peaks as shown in the last graph in Figure 5. Furthermore, there are many peak values side by side. This can have impact on the result of measurement and it is fixed by setting MinPeakDistance = 10 as a parameter of the function findpeaks in Matlab. Information about the *z*-component of a magnetic field signal is helpful (Figure 7 and Figure 8) to detect a car’s length more precisely.

The signals of the magnitude and the absolute z-component were presented for two cars in Figure 7 and Figure 8. As shown, the number of peaks can be different, the first and the last peak in the magnitude do not always correspond their analogues in the absolute *z*-component. Too high threshold level extends the risk for the omission of little peaks and can result in a lower length value. In turn, too low threshold level can adopt any interference peak or little peak in the noise as the first or the last one and it can increase a length value.

### 6.5. Method Based on the Amplitude and Time Normalization Using Linear Extrapolation or Interpolation (Method 4)

**Figure 9:**
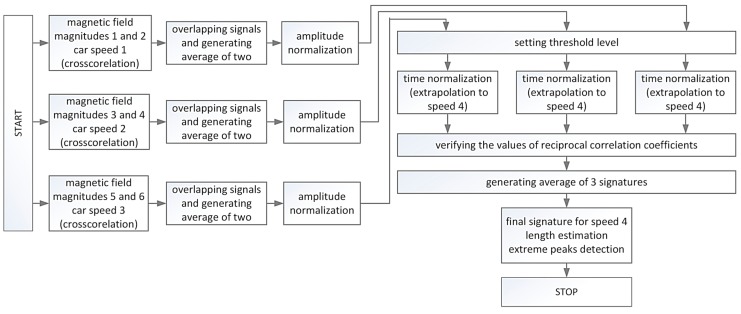
Algorithm for finding a car signature at one normalized speed basing on the magnetic field (data registered using one car and three drives at three different speeds).

This method is based on averaging six signals (three pairs), which were acquired at three speed values when the same car was driving by. The signals are stored in a database in order to obtain an average car magnetic signature at another, selected speed. Linear interpolation or extrapolation of a signature length and speed are used. Linear extrapolation is selected because it is based on three data points, e.g., cubic spline data extrapolation brings worse results in this case. It was verified that the results are satisfactory when the fourth, extrapolated point is on the line between three points or relatively close to an outer point (up to 15 km/h). Figure 9 shows the full signal processing algorithm. The limitation of this method is that it provides a good value of length between two extreme peaks only for similar (correlated) input datasets.

**Figure 10:**
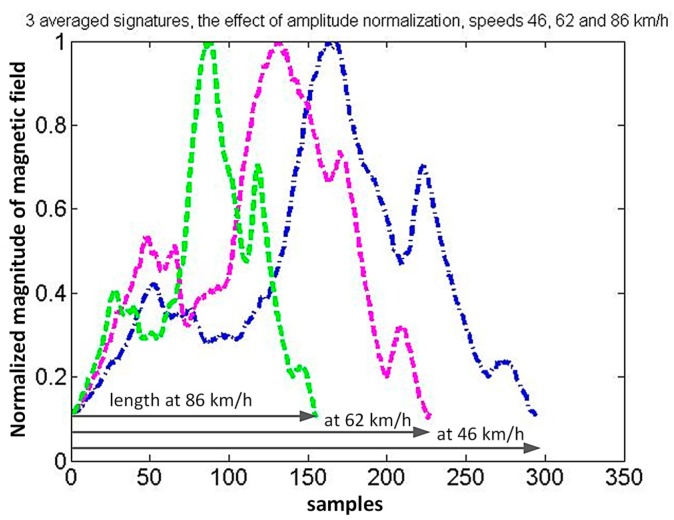
The effect of amplitude normalization and the estimation of three waveform lengths in samples.

**Figure 11:**
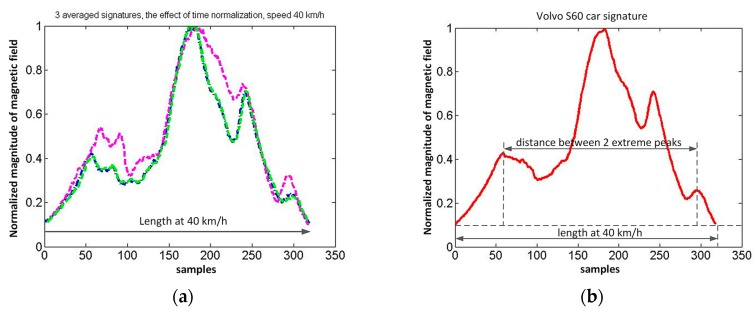
The effect of time normalization: (**a**) three waveforms have the same length after linear extrapolation to 40 km/h; (**b**) the average of three waveforms is a car signature, the distance between two extreme peaks is a measure of car length.

Figure 10 and Figure 11 present some selected signal processing steps to obtain the Volvo S60 signature. As a result of finding the three averaged waveforms, the amplitude normalizing and setting the common threshold value (0.1), three lengths in samples were obtained (Figure 10). Then the lengths in samples are sorted by decreasing velocity and the fourth length is determined. It is computed based on the ordered pairs of numbers (signature length, speed).

The linear extrapolation (interpolation) is used for this purpose. Time normalization (stretching) of three waveforms is made (Figure 11a). Stretching is based on the external input of speed value. It can be used to create a car classification system. After checking correlation coefficients, the final average signature is obtained and its length for the selected threshold. Correlation coefficients of signals from Figure 11a are *corr*_12_ = 0.957, *corr*_23_ = 0.959, *corr*_13_ = 0.998. The length at selected speed is computed in this way. Two extreme peaks are detected if all correlation coefficients are more than 0.94.

## 7. Results

The results of the research are presented step by step. At first (Table 1) cars were tested while driving on the road from the North to the South and from the South to the North. The cars of similar length and weight were driven at three different speed values. The Opel Vectra and Volvo S60 were driven centrally (C) along the lane axis. Lengths and distances between extreme peaks were computed using methods 1, 2 and 3. The results are based on computations with the use of the magnitude and the absolute *z*-component as input data. The threshold corresponding to 35 mG in method 1 was set. A number of 25 samples and 3SD was used in computations by means of the method 2 and treated as optimal settings.

The other results (Table 2) concern the cars driven on the road from the East to the West and from the West to the East. Tests were performed at similar speed values around 30 km/h. The Toyota Auris was driven centrally (C) along the lane axis or with 30 cm shift to the North (N) or to the South (S) in two directions. The same three methods were used.

Method 4 was used for cars which were driven centrally and non-centrally along a lane axis. Opel Vectra and Volvo S60 results are presented in the Table 3 at the same threshold level and with different combinations of speed values.

**Figure 12:**
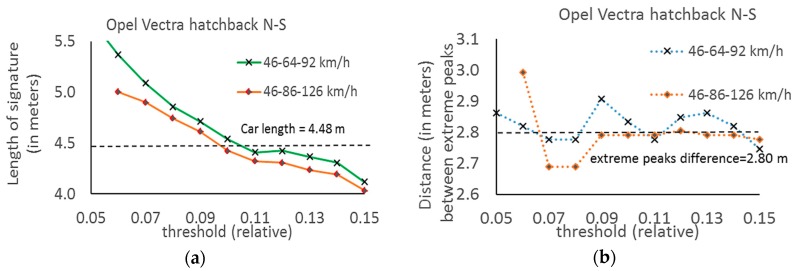
(**a**) Dependence of signature length on threshold—results obtained by means of method 4; (**b**) Dispersion of distance between extreme peaks with changing threshold—results obtained by means of method 4.

The results are sensitive to the threshold value (Figure 12a). This method can be helpful to choose one common threshold value for a group of cars. However, it is needed to perform more experiments in the future. As it is shown in Figure 12b the distance between extreme peaks does not depend on the threshold value in this method. Starting from the threshold at 0.05–0.08 level, the correlation coefficients were usually high and the final signature was reliable. This occurs when the signals are without “tails”, as in Figure 10.

In Table 4 the magnitude-based results of the experiment with the use of all cars, which were driven centrally along a road lane axis, are summarized. The data was averaged (10 repeats at different speed values for each type of a car and in different Earth directions), methods 1 and 3 were used (at threshold level 35 mG). The ratios between lengths (a magnetic signature length and a distance between two extreme peaks in a magnetic signature) and a real car’s length were calculated. The ratios are always less than 1 and depend on the car brand and the Earth direction, the ratio 1 varies from 0.64 to 0.90 and ratio 3—from 0.58 to 0.92. The smallest errors in length estimations (on average) are achieved with fixed ratio values of 0.75. The maximum errors (for averaged data) are 15.91% and 22.04%. They are defined as modulus of the relative difference between an obtained result (at ratio equal to 0.75) and a real car’s length.

## 8. Conclusions

Mid-size class cars differ in signature shape, number of peaks, value of their amplitudes, which causes a problem for creating universal software for accurate length estimation. The passenger cars were tested in four different Earth directions, as shown in Table 1. The results based on the magnitude (N-S direction) for Opel Vectra are up to 1 m higher and for Volvo S60 up to 1.2 m higher using methods 1 and 2. The method 1 assures less dispersion of result values. The Volvo results are 0.5 m greater than their Opel analogues. The reason can be that Volvo is 0.1 m longer and it has 0.03 m less ground clearance than the Opel. Lengths calculated basing on the magnitude of signal are longer than the real lengths of cars. The lengths computed basing on the absolute *z*-component signal are usually shorter. Method 3, used to detect a distance between two extreme peaks, this makes less difference between magnitude-based and absolute *z*-based values. These values are greater for Volvo S60 and the reason is that the wheelbase and construction of car body are different.

The car signature lengths measured in the E-W, W-E and S-N directions are longer than in the N-S. The Toyota Auris is a shorter car than the Opel Vectra and Volvo S60 yet the results in Table 2 are greater than those in Table 1. The differences were also visible when the Toyota Auris was driven centrally along the lane axis, with a 30 cm shift to the North (N) or to the South (S) in the forward and reverse direction. Amplitudes of signals were about 50–70% higher when the car was driven with 30 cm shift to the North (the car was closer to the Earth’s north pole). In the same case, taking the method 1 into account as the most reliable, the magnitude-based and absolute *z*-based values in Table 2 are greater or equal.

The values obtained when using the method 4 are similar to the equivalents when using the method 1 and 2. The measured distances between two extreme peaks (Table 3) were 0.1–0.2 m shorter in relation to values from the Table 1. It is a result of averaging several signals which are not always perfectly the same during driving at different speed values.

The final result of the car’s length comes out of combining the methods 1 and 3. It is assumed that car’s length is a value between a car signature length and a distance between two extreme peaks (Table 4). In this case the maximal error of estimated car’s length was 22%. In most cases the error values are lower when relating a real length to a car signature length (method 1).

Methods 1 and 2 are suitable mostly for a practical implementation. Method 1 is better when small distances appear among cars in a traffic situation. Method 2 is the preferred option when it is necessary to filter signals. Method 3 is unreliable because the number of peaks in the magnitude or *z*-component waveform varies from 3 to 7 in the case of sedans. Method 4 is proposed by the authors of this paper. This method can be used rather for creating a database with averaged signatures rather than for implementing it in a real-time speed and length detection system. The computation time in such a system should be as short as possible.

## Figures and Tables

**Table 1:** Signature length and two-extreme-peak distances (in meters) computed by means of three methods, abs z—absolute z.

Direction N-S	Method 1		Method 2		Method 3	
	Magnitude	abs z	Magnitude	abs z	Magnitude	abs z
Opel Vectra (C)	car length 4.481 m, wheelbase 2.639 m, ground clearance 0.16 m					
speed 1 = 46.1 km/h	5.117	4.116	5.233	5.299	2.917	3.000
speed 2 = 64.3 km/h	5.116	3.953	5.488	4.279	2.977	3.046
speed 3 = 86.5 km/h	5.125	3.899	5.062	3.900	2.937	3.033
mean	5.119	3.989	5.261	4.492	2.944	3.026
Volvo S60 (C)	car length 4.577 m, wheelbase 2.715 m, ground clearance 0.13 m					
speed 1 = 46.1 km/h	5.683	3.566	5.933	3.383	3.666	3.800
speed 2 = 62.9 km/h	5.477	3.773	5.772	3.273	3.659	4.182
speed 3 = 86.5 km/h	5.500	3.594	5.562	3.844	3.625	3.719
mean	5.553	3.641	5.756	3.513	3.650	3.900

**Table 2:** Signature length and two-extreme-peak distances (in meters) computed by means of three methods (car length 4.219 m, wheelbase 2.601 m, ground clearance 0.14 m).

**Direction E-W (Forward) Toyota Auris (C/S/N)**	**Method 1**		**Method 2**		**Method 3**	
	**Magnitude**	**abs z**	**Magnitude**	**abs z**	**Magnitude**	**abs z**
speed 1 = 27.4 km/h (C)	6.525	4.831	5.733	4.999	3.455	4.049
speed 2 = 35.0 km/h (S)	6.127	3.524	4.975	3.429	4.088	4.595
speed 3 = 32.9 km/h (N)	6.452	4.940	6.190	4.979	3.822	3.821
**Direction W-E (Reverse) Toyota Auris (C/S/N)**	**Method 1**		**Method 2**		**Method 3**	
	**Magnitude**	**abs z**	**Magnitude**	**abs z**	**Magnitude**	**abs z**
speed 1 = 28.5 km/h (C)	6.278	4.360	5.443	4.639	3.845	4.154
speed 2 = 37.4 km/h (S)	5.892	3.797	5.622	5.000	3.689	4.865
speed 3 = 31.8 km/h (N)	6.632	5.000	6.460	6.034	3.885	5.149

**Table 3:** Signature lengths and two-extreme-peak distances computed by means of method 4.

Direction N-S Normalized Speed 4 = 40 km/h	Method 4 (Magnitude)	Method 4 (Magnitude)
	Length of Signature (m)	Distance between Two Extreme Peaks (m)
Opel Vectra (C)	car length 4.481 m, wheelbase 2.639 m, ground clearance 0.16 m	
threshold 0.10, speed 1–3: 46, 86, 126 km/h	4.424	2.790
threshold 0.10, speed 1–3: 46, 64, 92 km/h	4.540	2.884
Volvo S60 (C)	car length 4.577 m, wheelbase 2.715 m, ground clearance 0.13 m	
threshold 0.10, speed 1–3: 46, 62, 86 km/h	4.597	3.412

**Table 4:** The average length measurement values yielded using the methods 1 and 3, real car lengths, ratios of the lengths, errors. Common ratio values 0. 75 were assumed for all car brands.

Car, Direction	Length (Met. 1) L1	Length (Met. 3) L3	Real Length RL	Ratio 1 RL/L1	Ratio 3 L3/RL	Result (Ratio 3 = 0.75)	Error (Ratio 3 = 0.75)	Result (Ratio 1 = 0.75)	Error (Ratio 1 = 0.75)
	m	m	m	-	-	m	%	m	%
O. Vectra, N-S	5.023	2.932	4.481	0.893	0.654	3.909	12.75	3.767	15.91
O. Vectra, S-N	6.168	3.269	4.481	0.727	0.730	4.358	2.72	4.626	3.26
Volvo S60, N-S	5.553	3.650	4.570	0.823	0.799	4.867	6.49	4.165	8.86
T. Avensis, E-W	6.887	4.153	4.765	0.693	0.872	5.537	16.21	5.165	8.40
T. Avensis, W-E	6.948	4.167	4.765	0.686	0.875	5.556	16.60	5.211	9.37
S. Superb, E-W	6.181	3.046	4.838	0.783	0.630	4.061	16.05	4.636	4.18
Lexus RX, E-W	6.385	4.366	4.770	0.747	0.915	5.821	22.04	4.789	0.39
